# Efficacy in urinary symptom burden, psychological distress, and self-efficacy of education-enhanced interventions in prostate cancer patients: a systematic review and meta-analyses

**DOI:** 10.1007/s00520-023-07803-6

**Published:** 2023-05-16

**Authors:** Javier Martín-Núñez, Julia Raya-Benítez, Laura López-López, Andrés Calvache-Mateo, Alejandro Heredia-Ciuró, Alba Navas-Otero, Marie Carmen Valenza

**Affiliations:** Faculty of Health Sciences, Department of Physiotherapy, University of Granada, Av. De La Ilustración, 60, 18016 Granada, Spain

**Keywords:** Urinary symptom burden, Psychological distress, Self-efficacy

## Abstract

**Background:**

Worldwide, prostate cancer is both the second-most diagnosed cancer and most common solid tumor in men. Prostate cancer patients present with a symptom burden that is compounded by the impact of medical oncology treatment, affecting different domains of their perceived health status. Education active techniques are a key role in chronic disease to increase participation in their recovery.

**Purpose:**

The purpose of the current review was to examine the efficacy of education-enhanced in urinary symptom burden, psychological distress, and self-efficacy in patients diagnosed with prostate cancer.

**Methods:**

A wide search of the literature was conducted for articles from their inception to June 2022. Only randomized controlled trials were included. Data extraction and methodologic quality assessment of the studies were carried out by two reviewers. We previously registered the protocol of this systematic review on PROSPERO (CRD42022331954).

**Results:**

A total of six studies were included in the study. After education-enhanced intervention showed significant improvements in any of perceived urinary symptom burden, one in psychological distress, and one in self-efficacy in the experimental group. The meta-analysis showed that education-enhanced interventions have a significant effect on depression.

**Conclusion:**

Education-enhanced could have positive effects on urinary symptom burden, psychological distress, and self-efficacy in prostate cancer survivors. Our review was unable to demonstrate the best timing to apply education-enhanced strategies.

## Introduction

Worldwide, prostate cancer is both the second-most diagnosed cancer and most common solid tumor in men [1, 2]. Estimated 1,414,259 cases and 307,000 deaths, becoming the fifth leading cause of cancer death in men [2, 3]. In addition, it is estimated to grow to almost 2.3 million new cases by 2040 due to the growth and aging of the population [2]. 

Prostate cancer is a commonly diagnosed cancer, and due to the large number of new prostate cancer diagnoses each year, millions of prostate cancer survivors are present worldwide [1]. These survivors present with a symptom burden that is compounded by the impact of medical oncology treatment, affecting different domains of their perceived health status (e.g., urinary symptoms, sexual function symptoms, bowel incontinence, psychological distress, and self-efficacy) [4, 5].

Among the high symptom burden of prostate cancer survivors, there is a high prevalence of symptoms related to sexual and urinary function. The presence of high symptom burden as well as specific factors of the disease and treatment influence the likelihood of experiencing psychological distress [6]. Among psychological symptoms, depression in prostate cancer patients is often an unidentified and underdiagnosis factor [7].

Additionally, prostate cancer survivors who have received androgen deprivation therapy presented a higher risk of developing depression than other therapies [8]. Also, those individuals experiencing depressive symptoms had lower self-efficacy [9]. A key role in chronic disease is to increase active participation in their recovery and self-management: a comprehensive approach to the management of chronic [10, 11] (this suggests the need to implement education active strategies in prostate cancer survivors. Those techniques can improve the person’s quality of life by removing the behavioral barriers that may get in the way of those improvements, achieving lasting generalization of both quality of life and behavioral improvements [12]. Among those techniques, education enhanced may be defined as the application of biomedical techniques to increase educational skills. These techniques include psychological strategies, support through clinical action plans or equipment, and even social support or the practice of self-management activities, among others. In this way, it allows patients to perform a comprehensive and active functional assessment of their own behavior [12].

To the best of our knowledge, there are no previous reviews that analyze the effects of education-enhanced programs on prostate cancer patients. Thus, the purpose of the current review was to examine the efficacy of education-enhanced in urinary symptom burden, psychological distress, and self-efficacy in patients diagnosed with prostate cancer.

## Methods

A systematic review and meta-analyses were performed to identify randomized clinical trials reviewing the effects of education-enhanced in psychological distress, symptom burden, and perceived self-efficacy. The guidelines of the Preferred Reporting Items for Systematic Reviews and Meta-Analyses (PRISMA) were used to achieve the systematic review. The Cochrane Collaboration guidelines for reviewing interventions were also closely followed. We previously registered the protocol of this systematic review on PROSPERO (CRD42022331954).

### Search strategy

A wide search of the literature was conducted for articles indexed on Pubmed, Scopus, and Web of Science databases from their inception to June 2022. A MEDLINE search strategy was developed based on the examination of keywords used in existing systematic reviews, such as the terms: “prostate,” “cancer,” “education,” “urinary symptom,” “psychological distress,” and “self-efficacy,” as well as comprehensive review of MeSH terms, expert guidance, and specialist review. Additionally, we screened the reference lists of relevant reviews related to the term and considered non-English language studies for inclusion if the translation was possible.

Articles were included if they met the following criteria: (1) prostate cancer survivors; (2) education-enhanced interventions; (3) education-enhanced had to be compared to an isolated education intervention; (4) urinary symptom burden, psychological distress, and self-efficacy were included in the outcomes; (5) only randomized control trials were included.

Two researchers carried out a search process that included removing duplicates and screening titles, abstracts, and eligible full texts. Also, the researchers independently performed the literature search, and disagreements were resolved through a consensus discussion with a third independent investigator to reduce the selection bias potential.

After selected articles data extraction and methodological assessment were carried out. Methodological quality assessment was evaluated by The Downs and Black Checklist, [13] one of the most used methodological quality assessment scales for randomized clinical trials. This tool consists of 27 items, including five subscales, which are: reporting, external validity, internal validity (study bias and confounding), selection bias, and study power. Poor quality is considered when a score of 14 or less is achieved, fair quality between 15 and 19, good between 20 and 25, and excellent study when it is higher or equal to 26 [14, 15].

Cochrane Risk of Bias Tool was used to assess the risk of bias for randomized controlled trial. A total of 6 subscales make up this tool subscales (selection bias, performance bias, detection bias, attrition bias, reporting bias, and another bias) [16]. The methodological quality depends on the risk of each of the subscales: high quality (low risk in all domains), fair quality (high risk in one domain or two unclear domains), and poor quality (two or more unclear domains or there are important limitations that could invalidate the results) [17].

### Meta-analysis

Quantitative synthesis of studies presenting means and standard deviations of symptom burden, psychological distress, and self-efficacy was carried out using Review Manager 5 software (RevMan 5). Quantitative data, including the number of patients assessed, final mean values, and standard deviations for each treatment arm, was extracted to estimate the overall mean differences between experimental and control arms.

When the studies did not present sufficient data to calculate the effect size (e.g., no means provided, no standard deviation provided), the authors were contacted. We calculated missing standard deviations when *n p*-values or 95% confidence intervals were given via the embedded Review Manager calculator.

We assumed to measure the same underlying symptom or condition, and therefore standardized mean differences were used as all the scales. The overall mean effect sizes were estimated using random effect models or fixed effect models according to statistical heterogeneity I2 tests (for sizes of less than 50%, fixed effect models were used) [16]. We also undertook a visual inspection of the forest plots for outlier studies, explored sources of heterogeneity, and conducted sensitivity analyses by excluding trials that were at a high risk of detection or attrition bias.

## Results

**Fig. 1:**
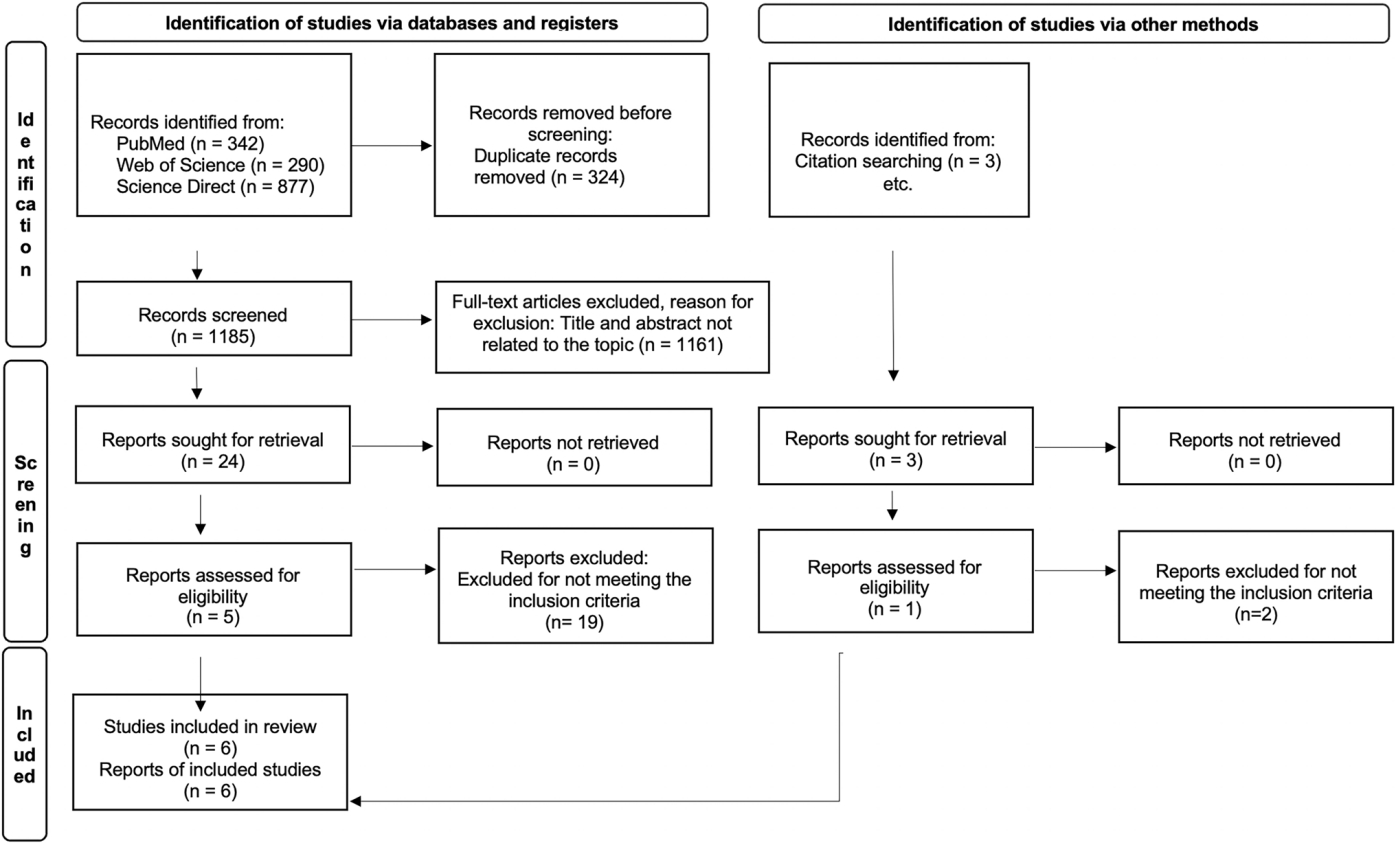
PRISMA flow chart of literature search and study selection

Figure 1 presents the process of the search, screening, and selection of studies. We collected a total of 1509 studies from the three electronic databases. In total, 1185 records remained after removing duplicates. A total of 24 articles were selected when we screened based on the title and abstract results. Of these 24 records, 19 articles were excluded due to the evaluation of the full text for not meeting the inclusion criteria, meeting 5 studies’ eligibility criteria. While 1 study was identified by other methods. Finally, a total of 6 studies [18–23] were included in the qualitative syntheses, and 5 studies were included in the quantitative syntheses [18–21, 23].

As shown in the flow chart, we finally included 6 studies in the review. Details about the characteristics of the studies are reported in Table 1. A total of 2 studies [18, 21] recruited prostate cancer patients, 1 study [23] included prostate adenocarcinoma patients, 1 study [22] included benign prostate hyperplasia patients, and 2 studies [19, 20] did not report the etiology. Of the included studies, 4 studies [20–23] did not report the stage of the patients; however, 1 study [18] included patients with early stages (I–III) and 1 study [19] included patients with advanced stages (III–IV).

**Table 1:** Characteristic of studies

Study	TNM cancer stage	Treatment status Timing	Sample	Sample age (years ± SD)	Quality assessment Downs and Black (risk of bias)
Tagai et al. (2021) [18]	Localized prostate cancer with no regional lymph node or distant metastasis, I–III	Posttreatment (radiotherapy and surgery)	EEG: 217 CG: 214	EEG: 63.8 ± 6.67 CG: 63.3 ± 7.51	21 (some concerns of bias)
Penedo et al. (2020) [19]	III–IV	Posttreatment (hormone therapy)	EEG: 95 CG: 97	EEG: 68.81 ± 8.54 CG: 68.87 ± 9.23	21 (some concerns of bias)
Skolarus et al. (2019) [20]	NR	Posttreatment (hormone therapy radiotherapy and surgery)	EEG: 278 CG: 278	EEG: 66.2 ± 7.1 CG: 67.2 ± 5.7	21 (some concerns of bias)
Galvao et al. (2017) [21]	Localized prostate cancer; NR	During treatment or posttreatment (hormone therapy radiotherapy and surgery)	EEG: 232 CG 231	EEG: 63.7 ± 7.6 CG: 65.1 ± 7.8	21 (high risk of bias)
Chen et al. (2012) [22]	Benign prostatic hyperplasia; NR	Posttreatment (hormone therapy)	EEG: 119 CG: 113	EEG: 71.32 ± 7.21 CG: 69.98 ± 8.33	19 (high risk of bias)
Carmack Taylor et al. (2006) [23]	Prostate adenocarcinoma; NR	During treatment (hormone therapy)	EEG 1: 46 EEG 2: 36 CG: 35	NR	24 (some concerns of bias)

*TNM*, TNM classification of malignant tumor; *SD*, standard deviation; *EEG*, education enhanced group; *CG*, control group; *NR*, non-reported

Regarding treatment status, 4 studies [18–20, 22] included patients after oncological medical treatment, 1 study [23] applied the intervention in medical treatment moment, and 1 study [21] included patients that were found during treatment or posttreatment. The oncological medical treatment present in the studies was radiotherapy and/or surgery in 3 studies [18, 20, 21] whereas hormone therapy was presented in 5 studies [19–23].

A total of 1991 prostate cancer patients have been included in this review, with the mean age of the participants ranging between 63.7 ± 7.6 and 71.32 ± 7.21 in the experimental group and 63.3 ± 7.51 to 70.39 ± 5. The studies’ quality scores ranged from 19 to 24. When the Cochrane Risk of Bias Assessment was applied, 4 studies presented fair quality [18–20, 23] and 2 studies presented poor quality [21, 22].

Details about the intervention and results are reported in Table 2.

**Table 2:** Studies of the effectiveness education-enhanced intervention

Study	Education enhanced interventions	Program (week) Frequency (days/week) Dose (total minutes)	Outcomes	Results
Tagai et al. (2021) [18]	• Education • Provision of equipment and agreement on specific clinical action plans and/or rescue medication	24; NR; NR	• Urinary symptom burden (EPIC) • Self-efficacy (TSEFRE; T13-iSEFSCS)	EEG improved significantly in urinary incontinence, urinary irritation, and sexual function with respect to baseline moment EEG improved significantly in urinary incontinence, urinary irritation, and sexual function compared to CG
Penedo et al. (2020) [19]	• Education • Training/rehearsal for practical self-management activities and psychological strategies	10; 1; 90	• Cancer-related anxiety (MAX-PC) • Cancer-Specific distress (IES-R) • Depression (PROMIS) • Perceived stress (PSS) • Positive affect (ABS) • Urinary symptom burden (EPIC) • Stress management skills self-efficacy (MOCS)	EEG improved significantly in sexual function, self-efficacy, and cancer related anxiety with respect to baseline moment EEG improved significantly in sexual function, self-efficacy, and cancer related anxiety compared to CG
Skolarus et al. (2019) [20]	• Education • Provision of agreement on specific clinical action plans and/or psychological strategies	16; NR; NR	• Urinary Symptom burden (EPIC) • Self-efficacy (PEPPI)	EEG improved significantly in incontinence, irritative, and obstructive domains with respect to baseline moment EEG improved significantly in incontinence, irritative, and obstructive domains compared to CG
Galvao et al. (2017)^21^	• Education • Provision of equipment, easy access to advice or support when needed and psychological strategies	24; NR; 60	• Psychological distress (BSI) • Urinary symptom burden (EPIC)	EEG improved significantly in psychological distress with respect to baseline moment No significant differences compare to control group
Chen et al. (2012) [22]	• Education • Training/ rehearsal for practical self-management activities • Social support	24; NR; NR	• Urinary symptom burden (IPSS)	EEG improved significantly in symptom burden with respect to baseline moment EEG improved significantly symptom burden compared to CG
Carmack Taylor et al. (2006) [23]	EEG 1: • Education • Provision of agreement on specific clinical action plans and/or rescue medication and practical self-management activities EEG 2: • Education • Provision of agreement on specific clinical action plans and/or rescue medication • Social support	24; 1–2; 1800	• Anxiety (STAI) • Depression (CES-D) • Self-efficacy	EEG1 and EEG2 improved significantly in self-efficacy respect to baseline moment EEG1 and EEG2 improved significantly in self-efficacy compared to CG

*NR*, non-reported; *EPIC*, Expanded Prostate Cancer Index Composite; *TSEFRE*, The Self-efficacy for Re-entry Scale; *T13-iSEFSCS*, The 13-Item Self-efficacy for Symptom Control Scale; *EEG*, education enhanced group; *CG*, control group; *MAX-PC*, The Memorial Anxiety Scale for Prostate Cancer; *IES-R*, The 22-Item Impact of Event Scale–Revised (IES-R); *PROMIS*, Patient-Reported Outcome Measurement Information System; *PSS*, Perceived Stress Scale; *ABS*: Affect Balance Scale; *MOCS*, measure of current status; *PEPPI*, perceived efficacy in patient-physician interactions; *BSI*, brief symptom inventory; *IPSS*, International Prostate Cancer Score; *STAI*, Scale of the State Trait Anxiety Inventory; *CES-D*, Centers for Epidemiologic Studies Depression

The components of the education-enhance programs were heterogeneous: provision of agreement on specific clinical action plans and/or psychological strategies realized in 3 studies, [18, 20, 23] psychological strategies realized in 3 studies too, [19–21] provision of equipment realized in 2 studies, [18, 21] rescue medication, [18, 23] training/rehearsal for practical self-management activities realized in 2 studies, [19, 22] social support realized in 2 studies [22, 23] and access to advice on support when needed realized in 1 study [21]. In relation to control group, all of them received education and usual care as an intervention.

Regarding the dose of education-enhanced, the frequency of intervention was expressed in only two studies and ranged from 1 to 2 days per week, and the intervention time ranged from 60 min to as much as 1800 min.

The outcomes explored in this review were urinary symptom burden, which was evaluated by 5 of the 6 studies [18–22], self-efficacy, which was evaluated in 4 studies [18–20, 23], and psychological distress, which was evaluated in 3 studies [19, 21, 23].

After experimental intervention, 4 studies [19, 20, 22] showed significant improvements in any of perceived urinary symptom burden in the experimental group concerning baseline and compared to the control group. In relation to self-efficacy, only 1 study [23] improved significantly in the experimental group concerning baseline compared to the control group. In terms of psychological distress, only 1 study [20] showed improvements in anxiety in the experimental group concerning baseline and compared to the control group and 1 study^22^ improved significantly only with respect to baseline moment.

### Results obtained in meta-analysis

**Fig. 2:**
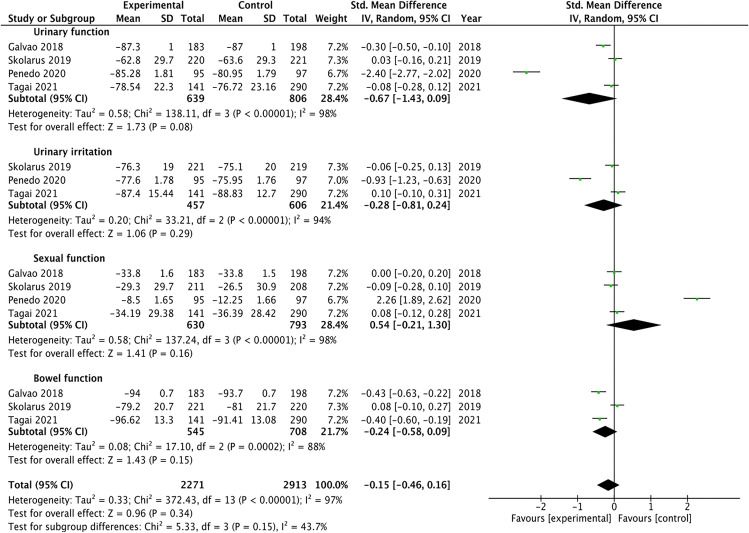
Meta-analysis: forest plot illustrating changes in urinary symptom burden. SD: Standard Deviation; CI: Coefficient Intervale

Figure 2 presents the results of the meta-analysis for urinary symptom burden of patients diagnosed with prostate cancer, divided by the different symptom. Although some symptoms showed results in favor of education-enhanced interventions compared to control group, none showed significant improvement in symptoms: urinary function (MD =  − 0.67, 95%, CI =  − 1.43, *P* = 0.08), urinary irritation (MD =  − 0.28, 95%, CI =  − 0.81, *P* = 0.29), sexual function (MD =  − 0.54, 95%, CI =  − 0.21, *P* = 0.16), and bowel function (MD =  − 0.24, 95%, CI =  − 0.58, *P* = 0.15). In the same lane, with respect to total urinary symptom burden measure reported, the pooled mean difference (MD) did not show significant overall effect of education-enhanced interventions compared with control group (MD =  − 0.15, 95%, CI =  − 0.46, *P* = 0.15). The results show heterogeneity, detecting significant variability of *I*^2^ = 97%.

**Fig. 3:**
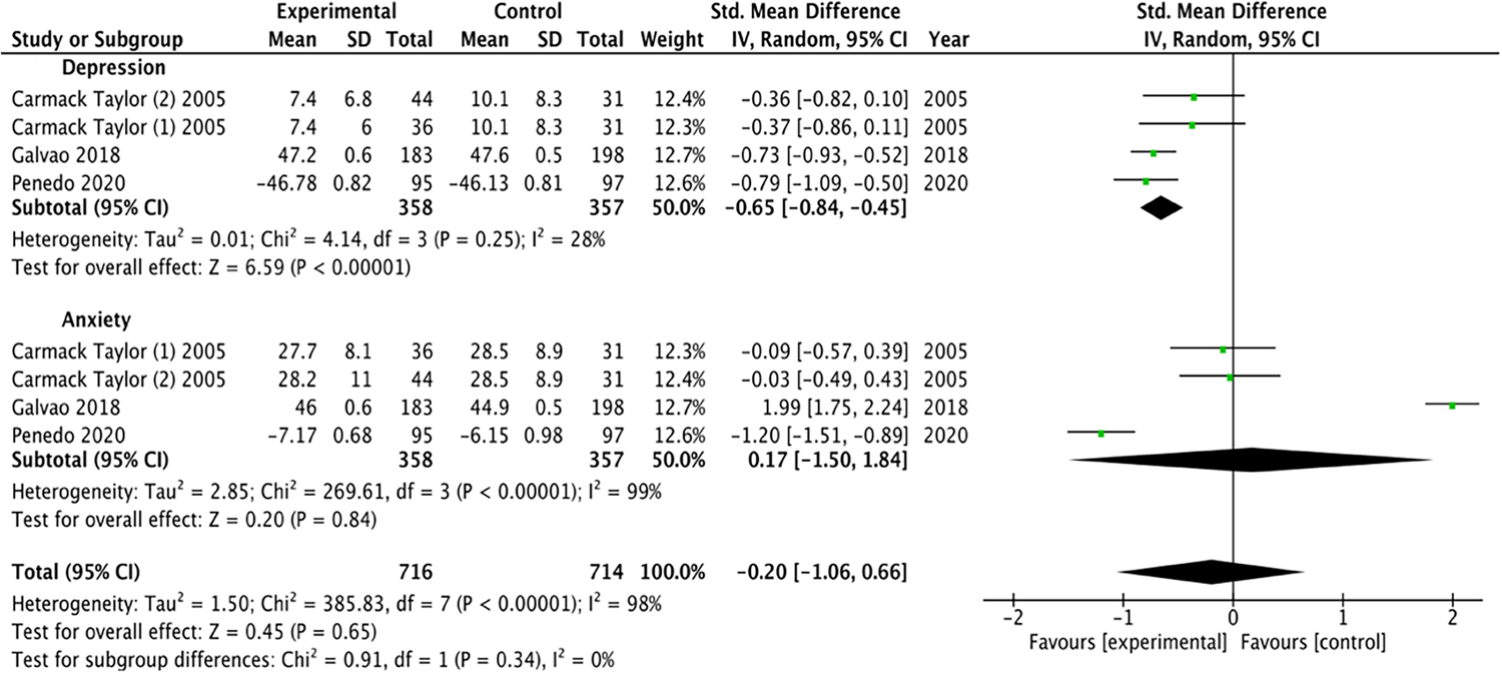
Meta-analysis: forest plot illustrating changes in psychological distress. SD: Standard Deviation; CI: Coefficient Intervale

Figure 3 presents the results of the meta-analysis of psychological distress of patients diagnosed with prostate cancer, divided by the different psychological disorders. For depression disorders, the pooled mean difference (MD) showed a significant overall effect of education-enhanced interventions compared to control group (MD =  − 0.65, 95%, CI =  − 0.84, *P* < 0.00001). For anxiety disorders, the pooled mean difference (MD) did not show significant overall effect of education-enhanced interventions compared to control group (MD = 0.17, 95%, CI =  − 1.50, *P* = 0.84). With respect to total psychological distress measure reported, the pooled mean difference (MD) did not show significant differences in favor of education-enhanced interventions compared to control group (MD =  − 0.20, 95%, CI =  − 1.06, *P* = 0.34). The results showed high heterogeneity, detecting significant variability of *I*_2_ = 98% that was not attributable to chance.

## Discussion

The aim of this current review was to examine the efficacy of education-enhanced interventions in urinary symptom burden, psychological distress, and self-efficacy in patients diagnosed with prostate cancer. However, our results should be interpreted with caution due to the number of education strategies implemented and dose of experimental intervention in the studies analyzed. Previous reviews showed results in agreement with ours, which suggest that education-enhanced interventions lead to beneficial effects in urinary symptom burden and psychological distress in different etiologies of cancer [24, 25]. In line with our results, education-enhanced seems to improve urinary symptom burden and psychological distress in patients diagnosed with cancer.

In relation to the urinary symptom burden, urinary function and urinary irritation only showed significant improvements in some studies [18, 20, 22], while sexual and bowel function had less impact in the included studies. Previous studies showed that urinary incontinence and function were a substantial enough problem after medical oncology treatment (especially after radiotherapy) [26, 27]. As for the meta-analysis, although no statistically significant improvements were found, improvements were shown in all variables except bowel function. As indicated by the studies of Merrick and Elshaikh [28, 29], the use of alpha-blockers improves urinary flow and irritation. In our case, all the studies included hormone therapy as a medical oncological treatment, except for the study of Tagai et al. [18].

With respect to psychological distress, anxiety and depression were both the most common mental disorders among prostate cancer patients in the included studies [18, 21, 23]. Previous research has observed that prostate cancer survivors included in their studies also presented anxiety and depression as the most common psychological disorders [8, 30]. Moreover, the studies of Sharp [31] and Occhipinti [32] associated a high urinary symptom burden with the likelihood of impaired psychological well-being. Similarly, low levels of self-efficacy interfere with the development of psychological stress [9]. The association of these variables suggests an improvement after the intervention.

## Limitations

Several limitations we have to take into account in this study. No homogeneity was observed in the stage of prostate cancer patients, which has an impact on the classification of the results. Some studies included a sample of patients with prostate cancer during medical oncology treatment as well as after undergoing treatment, which should be taken into account when interpreting the results. Neither the beginning nor the complete duration of the medical oncological treatment is specifically expressed, which has a great impact on the development of signs and symptoms in these patients; therefore, it would be of interest that future studies include this information. In addition, despite reviewing multiple electronic databases of published and unpublished studies, it is possible that some articles may have been omitted. Finally, it was not possible to perform a meta-analysis with the follow-up data.

## Conclusion

In conclusion, education enhanced could have positive effects on urinary symptom burden and psychological distress in prostate cancer survivors. Our review was unable to demonstrate the best timing to apply education-enhanced strategies. Although the meta-analysis showed results in favor of the experimental group for both the perception of urinary symptoms burden and psychological distress, only statistical differences were shown in the experimental group in depression.

## Data Availability

Not applicable.
